# Suppression of CD80 Expression by ICP22 Affects Herpes Simplex Virus Type 1 Replication and CD8^+^IFN-γ^+^ Infiltrates in the Eyes of Infected Mice but Not Latency Reactivation

**DOI:** 10.1128/JVI.01036-21

**Published:** 2021-09-09

**Authors:** Harry H. Matundan, Shaohui Wang, Ujjaldeep Jaggi, Jack Yu, Homayon Ghiasi

**Affiliations:** a Center for Neurobiology and Vaccine Development, Ophthalmology Research, Department of Surgery, Cedars-Sinai Burns and Allen Research Institute, CSMC-SSB3, Los Angeles, California, USA; Lerner Research Institute, Cleveland Clinic

**Keywords:** CD80 promoter, mapping, virus replication, cornea, transfection, luciferase, HSV-1, ICP22

## Abstract

Previously, we reported that herpes simplex virus type 1 (HSV-1) ICP22 binds to the CD80 promoter and suppresses its expression *in vitro* and *in vivo*. To better understand the impact of ICP22 binding to CD80 on HSV-1 infectivity and pathogenicity, we mapped the region of ICP22 required to bind the CD80 promoter to a 40-amino-acid (aa) region of ICP22. We constructed a recombinant HSV-1 expressing a truncated form of ICP22 that lacks these 40 aa, which does not bind to the CD80 promoter (KOS-ICP22Δ40) and retains the ability to replicate efficiently in rabbit skin cells, in contrast to ICP22-null virus. The replication of this recombinant virus *in vitro* and *in vivo* was higher than that of the ICP22-null virus, but virus replication kinetics were lower than those of the wild-type (WT) control virus. Similar to ICP22-null virus, the KOS-ICP22Δ40 mutant virus increased CD80 expression in dendritic cells (DCs) and interferon gamma (IFN-γ) expression in CD8^+^ T cells but not CD4^+^ T cells in infected mouse corneas. In contrast to the significantly reduced virus replication in the eyes of ocularly infected mice, the levels of latency reactivation were similar between KOS-ICP22Δ40 virus and WT virus. Thus, blocking ICP22 binding to the CD80 promoter using a recombinant virus expressing a truncated ICP22 that lacks CD80 promoter binding appears to reduce virus replication and enhance CD8^+^IFN-γ^+^ infiltrates in corneas of infected mice, with no effect on latency reactivation.

**IMPORTANCE** Direct binding of HSV-1 ICP22 to the CD80 promoter downregulates the expression of the costimulatory molecule CD80 but not CD86. In this study, we fine mapped the region of ICP22 required for binding to the CD80 promoter and constructed a recombinant virus containing a deletion in ICP22 that failed to bind to the CD80 promoter. This recombinant virus replicated less efficiently *in vitro* and *in vivo* than did the WT control virus, although CD80-expressing CD11c^+^ cells and IFN-γ-expressing CD8^+^ T cells were increased. Interestingly, the levels of latency and reactivation in the two viruses were similar despite lower virus replication in the eyes of infected mice. Therefore, blocking the interaction of ICP22 with the CD80 promoter could be used to temper the immune response.

## INTRODUCTION

Herpes simplex virus type 1 (HSV-1) infections that induce eye disease ultimately lead to blindness and are among the most frequent and serious viral eye infections in the United States (1–3). Following primary ocular infection and virus replication, HSV-1 establishes latency in the trigeminal ganglia (TG) of infected individuals (4, 5). Serious eye infections occur following viral reactivation from latency (6, 7). Thus, identifying mechanisms that lead to latency is of great clinical significance. CD4^+^ and CD8^+^ T cells are both involved in protecting as well as inducing HSV-1-induced eye disease depending on the strain of virus or mice used (8–13). Full activation of CD4^+^ and CD8^+^ T cells requires antigen (Ag)-specific signals and costimulatory signals (14).

Recently, we reported that ocular infection of mice with wild-type (WT) HSV-1 suppresses the expression of the costimulatory molecule CD80, but not CD86, in corneas of ocularly infected mice (15). A variety of cell types, including B cells, macrophages, dendritic cells (DCs), and T cells, express CD80 (16–19), and T cell activation requires the binding of CD80 to CD28 on T cells (20, 21). The use of CD80 (over CD86) can result in the development of a different immune response (22–28). We previously found that the binding of HSV-1 ICP22 to CD80 expressed on the surface of DCs suppresses CD80 expression (15). In contrast, a recombinant virus with a complete deletion of the *ICP22* gene failed to suppress CD80 expression despite significantly reducing virus replication both *in vitro* and *in vivo*, leading to enhanced eye disease (29). Therefore, we hypothesize that HSV-1 uses CD80 suppression as a mechanism of immune escape.

HSV-1 carries at least 85 genes (30), and HSV-1 replication is orchestrated by a cascade of three sets of genes (31, 32). We have shown that deletion of the *ICP22* gene, but not any of the other HSV-1 genes, represses CD80 expression by removing the ability of the ICP22 protein to directly bind to the CD80 promoter (15). The ability of ICP22 to bind to and suppress the CD80 promoter dampens the host immune response, allowing HSV-1 to partially escape immune surveillance, leading to reduced eye disease. Thus, *ICP22* may be a novel CD80 inhibitor that could be used therapeutically to modulate the immune response. The precise biological function of *ICP22* is unknown, but our published work suggests that mice infected with recombinant HSV-1 expressing CD80 have elevated levels of CD80 and CD8 and enhanced corneal scarring (CS) (15). We have also shown that the absence of ICP22 enhances eye disease in ocularly infected mice (29). Because the downregulation of CD80 and CD8 is required for virus infectivity, *ICP22* may be an HSV-1 survival mechanism to reduce the cytotoxic T lymphocyte (CTL) function of CD8, thus blocking cell lysis.

Since the complete deletion of ICP22 increases CD80 and CD8 expression in corneas of infected mice, leading to increased eye disease (15), the current study was designed to define the amino acid region of ICP22 required for binding to the CD80 promoter, generate a recombinant virus lacking these ICP22 amino acids, and determine whether the effect of this truncated virus on HSV-1 infectivity is similar to that of the ICP22-null virus *in vitro* and *in vivo*. We now have defined the amino acids required for ICP22 binding to the CD80 promoter and have deleted these amino acids in a recombinant virus (i.e., KOS-ICP22Δ40). Our results show that (i) in contrast to the parental virus and similar to WT KOS, KOS-ICP22Δ40 virus grew in rabbit skin (RS) cells; (ii) in contrast to WT KOS, KOS-ICP22Δ40 virus expresses a truncated form of ICP22; (iii) in contrast to WT KOS, KOS-ICP22Δ40 virus did not suppress the expression of CD80 on dendritic cells in the corneas of infected mice; (iv) KOS-ICP22Δ40 virus replication is intermediate to those of ICP22-null and WT viruses both *in vitro* and in the eyes of ocularly infected mice; (v) levels of CD8^+^ interferon gamma-positive (IFN-γ^+^), but not CD4^+^IFN-γ^+^, cells increased in the corneas of KOS-ICP22Δ40-infected mice compared with mice infected with the WT control virus; and (vi) latency reactivation of KOS-ICP22Δ40 and WT KOS was similar for both viruses.

## RESULTS

### Construction of ICP22 fragments to map the specific region of ICP22 that suppresses CD80 promoter activity *in vitro*.

**FIG 1:**
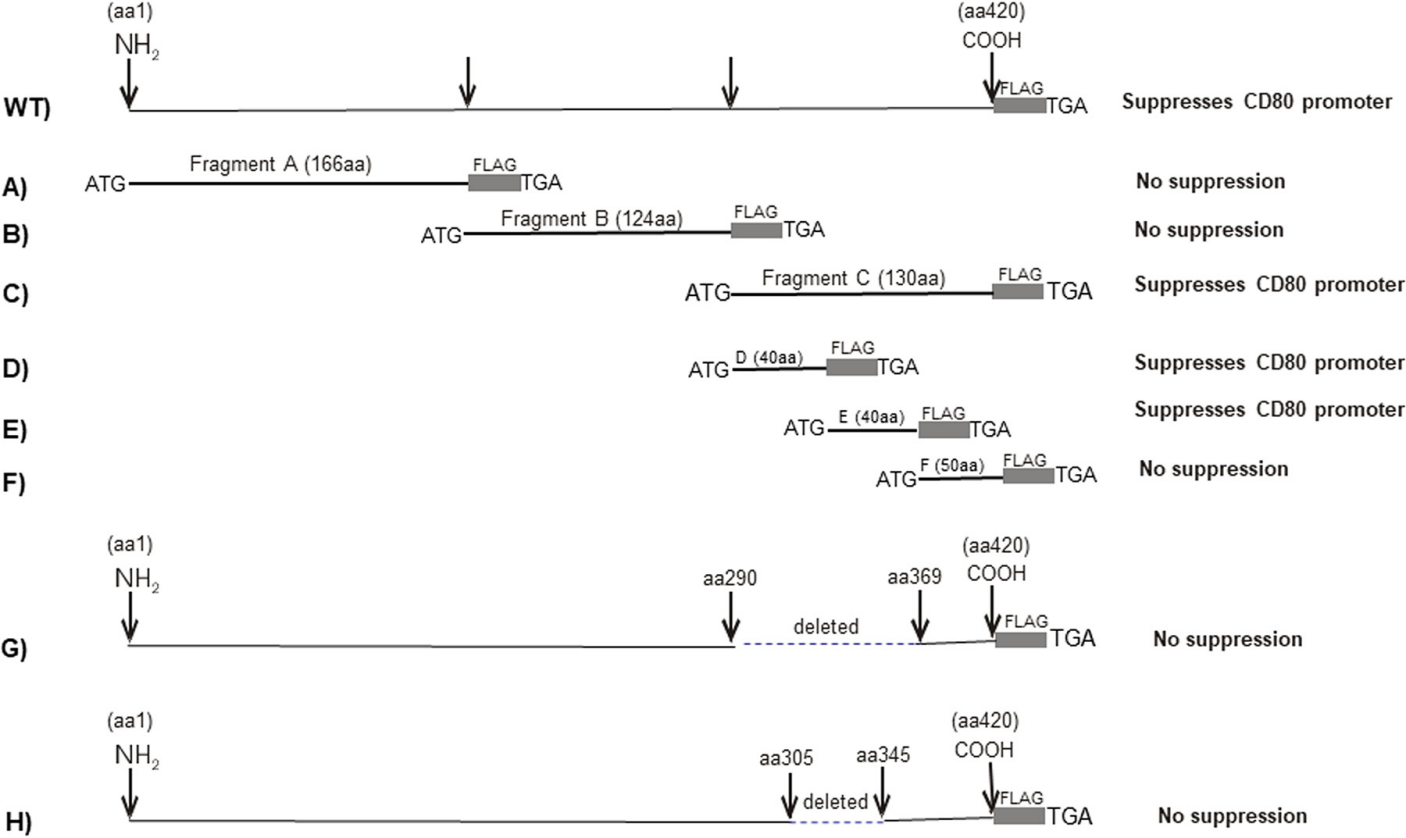
Schematic diagram of ICP22 constructs used to map ICP22 amino acids involved in binding to the CD80 promoter. The WT ICP22 protein (420 aa) is shown with an in-frame FLAG tag insertion (top). Each fragment contains an in-frame FLAG tag at the 3′ end. Each construct was inserted into the BamHI site of plasmid pcDNA3.1 as we described previously (15). The functional effect of each fragment on CD80 promoter activity is shown to the right of each fragment.

We have demonstrated that ICP22 binds to the CD80 promoter and that this binding suppresses CD80 expression *in vitro* and *in vivo* (15). Our previous study used a full-length *ICP22* gene construct with two in-frame copies of a FLAG tag at the 3′ end (Fig. 1) to assess binding to the CD80 promoter. Here, we refer to this construct as the WT (15). To extend our previous work, we constructed ICP22 fragments to map ICP22 amino acid regions that are required for binding to the CD80 promoter. A schematic diagram of ICP22 fragments used in this study is shown in Fig. 1. We first constructed three ICP22 fragments corresponding to amino acids (aa) 1 to 166 (fragment A, 166 aa [ICP22-A]), aa 166 to 290 (fragment B, 124 aa [ICP22-B]), and aa 290 to 420 (fragment C, 130 aa [ICP22-C]) (Fig. 1). These fragments were sequenced and expressed proteins of the expected size as determined by Western blotting using anti-FLAG antibody (not shown). These plasmids were used to map ICP22 amino acids that regulate CD80 expression as we described previously (15).

**FIG 2:**
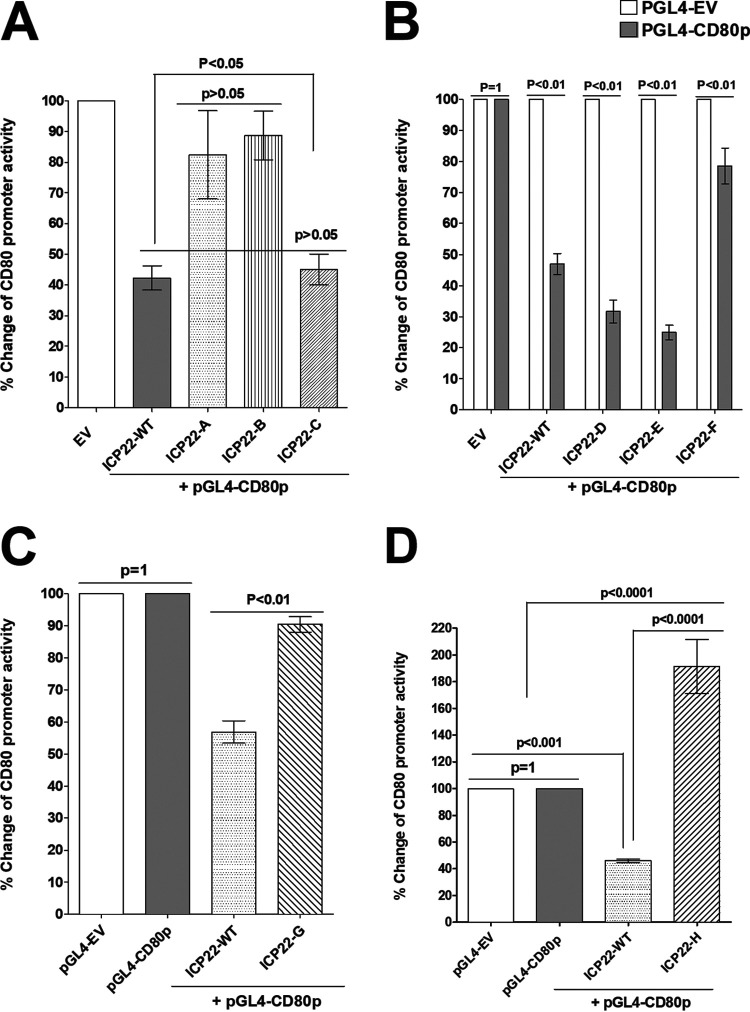
Mapping the amino acid region of ICP22 that suppresses the CD80 promoter. (A) Effect of *ICP22* gene fragments on CD80 promoter expression. Plasmid pGL4-EV or CD80 promoter (pGL4-CD80p) was cotransfected into 293 cells along with ICP22-A, ICP22-B, and ICP22-C plasmids. ICP22-WT (Fig. 1) was used as a control. The effect of ICP22 fragments on CD80 promoter activity was determined at 48 h posttransfection. Each point represents the mean ± SEM (*n* = 12) from three separate experiments. (B) Fine mapping of ICP22-C amino acids involved in suppressing CD80 promoter activity. The ICP22-C fragment was divided evenly into three fragments: ICP22-D, ICP22-E, and ICP22-F (Fig. 1). The pGL4-EV or pGL4-CD80p plasmid was cotransfected into 293 cells with pICP22-D, pICP22-E, or pICP22-F. ICP22-WT DNA was used as a control. CD80 promoter activity was determined at 48 h posttransfection. Each point represents the mean ± SEM (*n* = 9) from three separate experiments. (C) ICP22-G fragment repression of CD80 promoter activity. The ICP22-G fragment functionally lacks 79 aa of ICP22 (Fig. 1). Plasmid pGL4-EV or pGL4-CD80p was cotransfected into 293 cells along with ICP-WT or pICP22-G DNA to determine their effect on CD80 promoter activity at 48 h posttransfection. Each point represents the mean ± SEM (*n* = 9) from three separate experiments. (D) Effect of the ICP22-H fragment on suppression of CD80 promoter activity. The ICP22-H fragment functionally lacks 40 aa of ICP22 (Fig. 1). Plasmid pGL4-EV or pGL4-CD80p was cotransfected into 293 cells along with ICP-WT or pICP22-H DNA to determine their effect on CD80 promoter activity at 48 h posttransfection. Each point represents the mean ± SEM (*n* = 9) from three separate experiments.

pGL4-EV (control plasmid) or pGL4-CD80p DNA was transfected into HEK 293 (293) cells. The cells were also cotransfected with plasmids expressing ICP22 fragment A, B, or C, and their effect on CD80 promoter activity was determined by measuring luciferase activity 48 h later as we described previously (15). The effects of fragments A, B, and C on CD80 promoter activity are shown in Fig. 2A. Our results suggest that fragment A (Fig. 2A, ICP22-A) and fragment B (Fig. 2B, ICP22-B) did not suppress CD80 promoter activity compared to the empty vector (EV) control (Fig. 2A, EV). However, similar to WT ICP22 (Fig. 2A, ICP22-WT), fragment C (Fig. 2A, ICP22-C) suppressed CD80 promoter activity.

To further map the suppressive region of ICP22 within fragment C, we created three new ICP22 fragments by dividing fragment C into three fragments: D (Fig. 1D) (40 aa), E (Fig. 1E) (40 aa), and F (Fig. 1F) (50 aa). Each of these fragments had an ATG, a 2× FLAG tag, and a termination codon, and they all expressed proteins of the expected size (not shown). As described above, the three fragments were inserted into a pGL4 plasmid, and pGL4-EV was used as a control. Our results suggest that similar to full-length ICP22 (Fig. 2B, WT) and fragment C (Fig. 2A, ICP22-C), fragments D (Fig. 2B, ICP22-D) and E (Fig. 2B, ICP22-E), but not fragment F (Fig. 2B, ICP22-F), suppressed CD80 promoter activity. These results suggest that the suppressive part of ICP22 lies between ICP22 aa 290 and 369.

Based on fragments E and F, we created an ICP22 construct lacking 79 aa of ICP22 from aa 290 to aa 369 having an ATG, a 2× FLAG tag, and a termination codon (Fig. 1G). In contrast to full-length ICP22 (Fig. 2C, WT), ICP22-G did not suppress CD80 promoter activity (Fig. 2C). The promoter activities of ICP22-G and ICP22-WT were statistically significant (*P* < 0.05). Thus, the deletion of aa 290 to 369 eliminated the suppression of the CD80 promoter by ICP22 and is consistent with the suppressive region of ICP22 being within aa 290 to 369. To further map this activity based on fragment G and overlapping fragments E and F (Fig. 1), we created another construct lacking ICP22 aa 305 to 345 and having an ATG, a 2× FLAG tag, and a termination codon (Fig. 1H). This construct did not suppress CD80 promoter activity (Fig. 2D, ICP22-H), and the difference between ICP22-H and ICP22-WT was statistically significant (*P* < 0.0001). These results suggest that the region of ICP22 that suppresses CD80 promoter activity is located within ICP22 aa 305 to 345.

### Generation of KOS-ICP22Δ40 recombinant HSV-1 lacking ICP22 aa 305 to 345.

**FIG 3:**
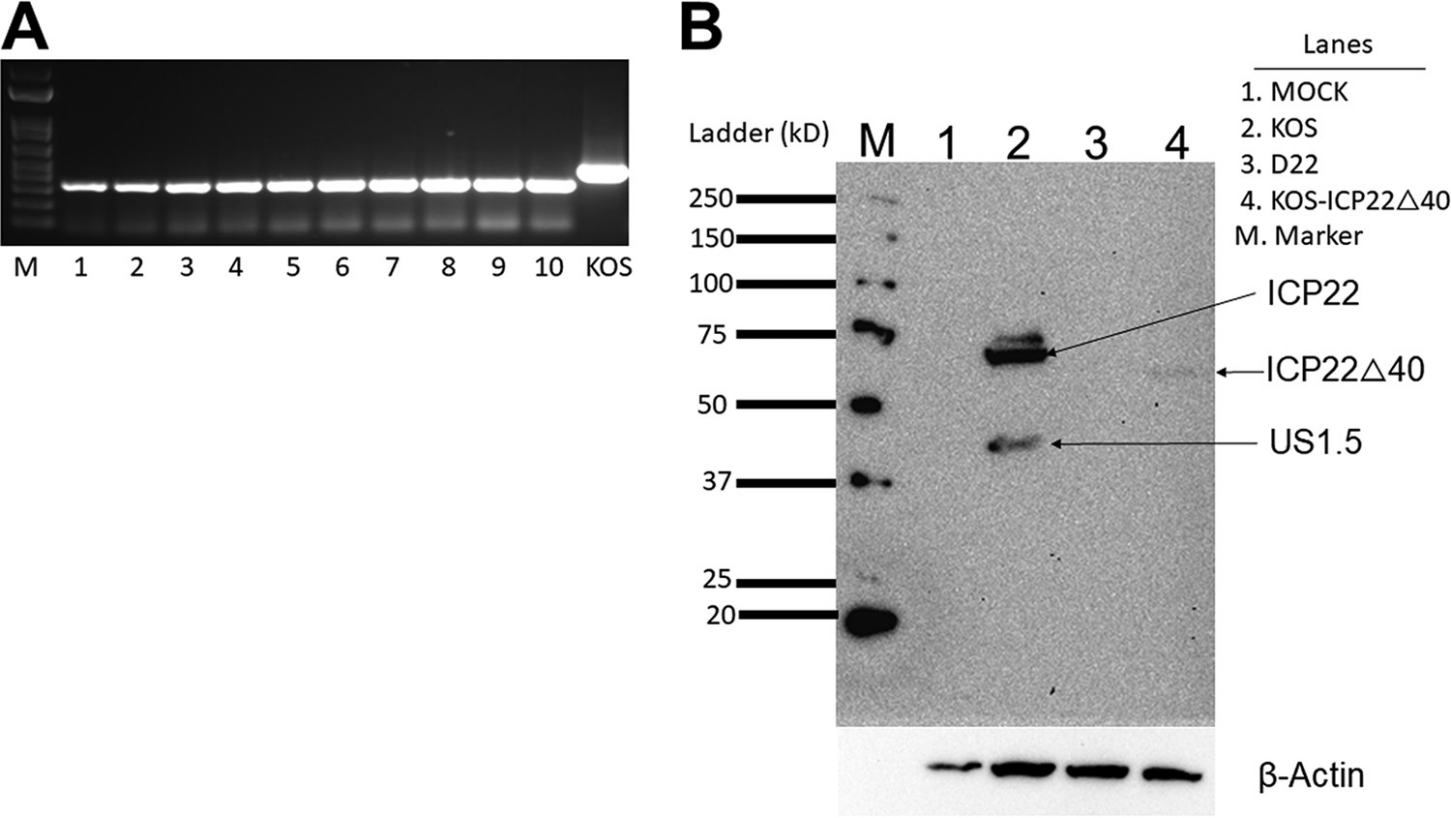
Construction of the pICP22Δ40 plasmid and KOS-ICP22Δ40 virus. The recombinant transfer vector plasmid contains HSV-1 nucleotides 132181 to 134460 with a 120-bp deletion in the *ICP22* gene. KOS-ICP22Δ40 virus was constructed from D22 virus by homologous recombination between D22 DNA and pICP22Δ40 plasmid DNA as described in Materials and Methods. (A) Isolation of the ICP22 truncated mutant. Ten plaques resulting from the cotransfection of D22 DNA and the pICP22Δ40 plasmid into RS cells were isolated. Subconfluent RS cell monolayers were infected with each plaque for 24 h. WT KOS was used as a positive control. Viral DNA was isolated, PCR was performed, and products were separated on an agarose gel. Lanes: M, marker; 1 to 10, mutant viruses; KOS, WT KOS. As expected, all transfection mixtures containing both D22 DNA and a pICP22Δ40 plasmid contained the truncated form of ICP22 (lanes 1 to 10), while ICP22 from WT KOS had a higher-migrating band (lane KOS). Plaques 9 and 10 were sequenced, and both contained the expected deletion. Plaque 9 (called KOS-ICP22Δ40) was verified by sequencing and used for subsequent studies. (B) Western blotting of KOS-ICP22Δ40-infected cells. 293 cells were infected with KOS, D22, or KOS-ICP22Δ40 at 1.5 PFU/cell for 5 h. Mock-infected cells were used as a control. Infected and mock-infected cells were harvested and lysed with RIPA buffer. For each lane, 40 μg of total whole-cell lysate protein was separated on a 10% SDS-PAGE gel. The blot was probed with anti-ICP22 antibody and anti-β-actin antibody. β-Actin was used as a loading control. Lanes: M, ladder; 1, mock infected; 2, KOS infected; 3, D22 infected; 4, KOS-ICP22Δ40 infected. Arrows indicate bands corresponding to ICP22 protein or the cross-reactive US1.5 in KOS-infected cells.

Because ICP22 aa 305 to 345 were shown to suppress CD80 promoter activity, we next asked whether this region also affects HSV-1 infectivity by generating a recombinant virus lacking ICP22 aa 305 to 345 for evaluation *in vitro* and *in vivo*. D22 recombinant virus lacking the entire *ICP22* gene was derived from the parental HSV-1 KOS strain (33), and we have evaluated its effect on CD80 expression *in vitro* and *in vivo* (29). Based on published HSV-1 KOS strain sequences (34), we synthesized a plasmid containing the *ICP22* gene with a deletion of 40 aa (aa 305 to 345) within ICP22 (pICP22Δ40). This plasmid also contained 463 bp of KOS sequence upstream of the ICP22 ATG and 554 bp of KOS sequence downstream of the ICP22 termination codon. We used D22 as the parental virus to generate KOS-ICP22Δ40 recombinant virus by cotransfecting pICP22Δ40 DNA and D22 DNA into RS cells as we described previously (47). Unlike KOS, D22 does not grow on RS cells; thus, we expected all generated viruses to contain the truncated form of the *ICP22* gene. We isolated DNA from 10 plaques and performed PCR on isolated DNAs and on the control, WT KOS. All 10 isolated plaques contained the truncated form of ICP22 (Fig. 3A). We sequenced plaques 9 and 10, and both had the expected deletion (not shown). Plaque 9 was propagated for subsequent studies with this deleted virus (KOS-ICP22Δ40). Thus, KOS-ICP22Δ40 virus is similar to WT KOS virus except for its 40-aa truncation within the *ICP22* gene.

To determine if KOS-ICP22Δ40 virus expresses the truncated form of ICP22, we used Western blot analysis with an anti-ICP22 antibody (Ab-413). Human embryonic kidney (HEK) 293 cells were infected with HSV-1 WT KOS, D22 having a complete deletion of ICP22, or KOS-ICP22Δ40 having a recombinant ICP22 with a 40-aa deletion or were mock infected. As expected, neither mock nor D22 expressed a detectable ICP22 product (Fig. 3B, lanes 1 and 3). Similar to previous reports (35, 36), 413 antibody detects ICP22 as well as a slower-migrating band corresponding to US1.5 in KOS-infected cells (Fig. 3B, lane 2). A fast-migrating band was detected in KOS-ICP22Δ40-infected cells (Fig. 3B, lane 4) compared to full-length ICP22 in KOS-infected cells (Fig. 3B, lane 2). Despite loading 40 μg of protein for each sample, the expression of the ICP22-Δ40 band in KOS-ICP22Δ40-infected cells (lane 4) was much fainter than WT ICP22 expression in KOS-infected cells (lane 2). Finally, US1.5, the shorter version of ICP22 visible in lane 2 of KOS-infected cells, was not detected in KOS-ICP22Δ40-infected cells, possibly due to its lower expression level (Fig. 3B, lane 4). The blot was stained for β-actin as a loading control, and β-actin expression levels were similar in the infected groups (Fig. 3B, β-actin). These results suggest that KOS-ICP22Δ40 virus expresses the truncated form of ICP22 but at significantly lower levels, possibly because it is less infectious than WT KOS *in vitro*. This led us to next evaluate the replication of KOS-ICP22Δ40 virus *in vitro* and *in vivo*.

### Replication of KOS-ICP22Δ40 virus in tissue culture.

**FIG 4:**
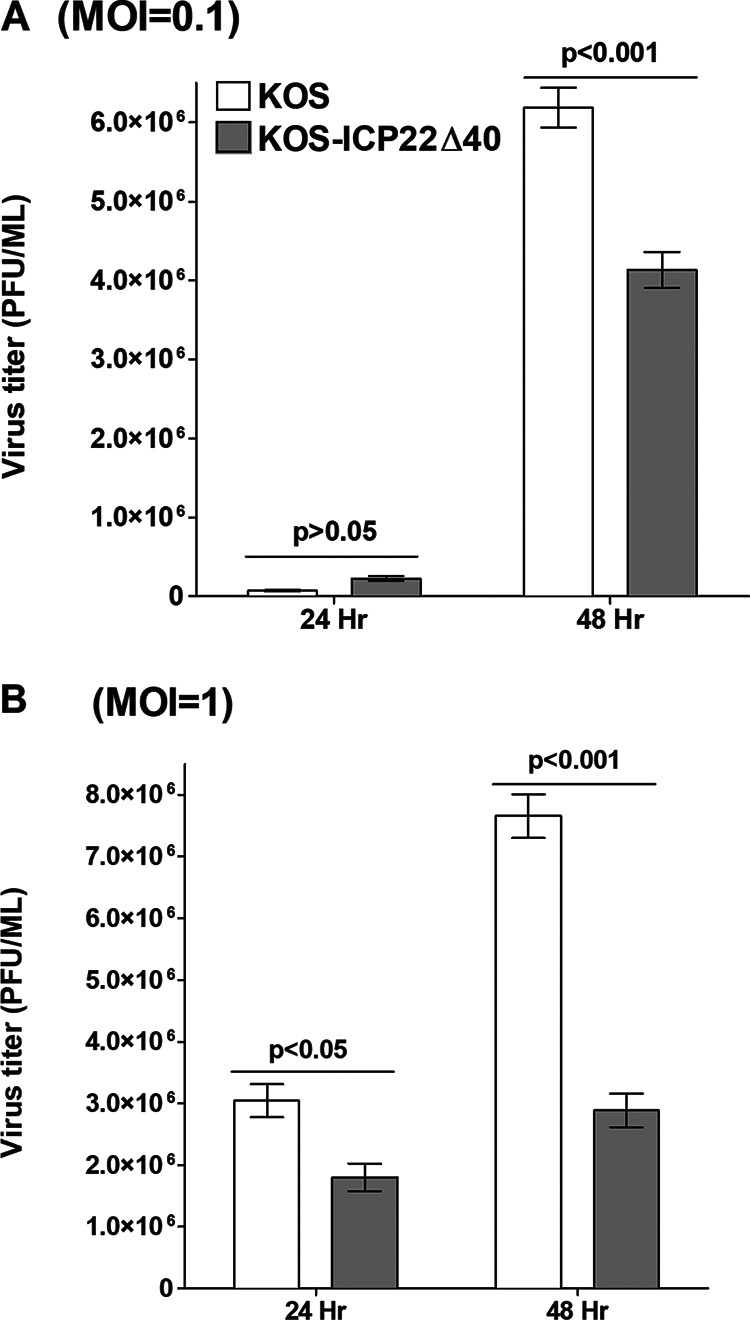
KOS-ICP22Δ40 replication in tissue culture. Subconfluent RS cell monolayers were infected with 0.1 or 1 PFU per cell of KOS-ICP22Δ40 or WT KOS for 24 and 48 h. Total virus was harvested at the indicated times postinfection by two cycles of freeze-thawing. Titers of each virus were determined at each time point by standard plaque assays on RS cells. Each point represents the mean ± SEM (*n* = 6). MOI, multiplicity of infection.

RS cells were infected with 0.1 or 1 PFU/cell of KOS-ICP22Δ40 virus or WT KOS. Cell monolayers were freeze-thawed at the indicated times, and the virus yield was determined as described in Materials and Methods. The levels of replication at 0.1 PFU/cell of KOS-ICP22Δ40 virus and WT KOS were similar at 24 h postinfection (p.i.) (*P* > 0.05) (Fig. 4A), but by 48 h p.i., the replication of WT KOS was significantly higher than that of the KOS-ICP22Δ40 mutant virus (*P* < 0.001) (Fig. 4A). At 1 PFU/cell, the replication of WT KOS was significantly higher than that of the KOS-ICP22Δ40 mutant virus at 24 h p.i. (*P* < 0.05) (Fig. 4B, 24 h) and 48 h p.i. (*P* < 0.001) (Fig. 4B). Similar results were obtained using Vero cells (not shown). Thus, the deletion of ICP22 aa 305 to 345 (40 aa) reduced or delayed virus replication in tissue culture.

### Virus titers in mouse tears.

**FIG 5:**
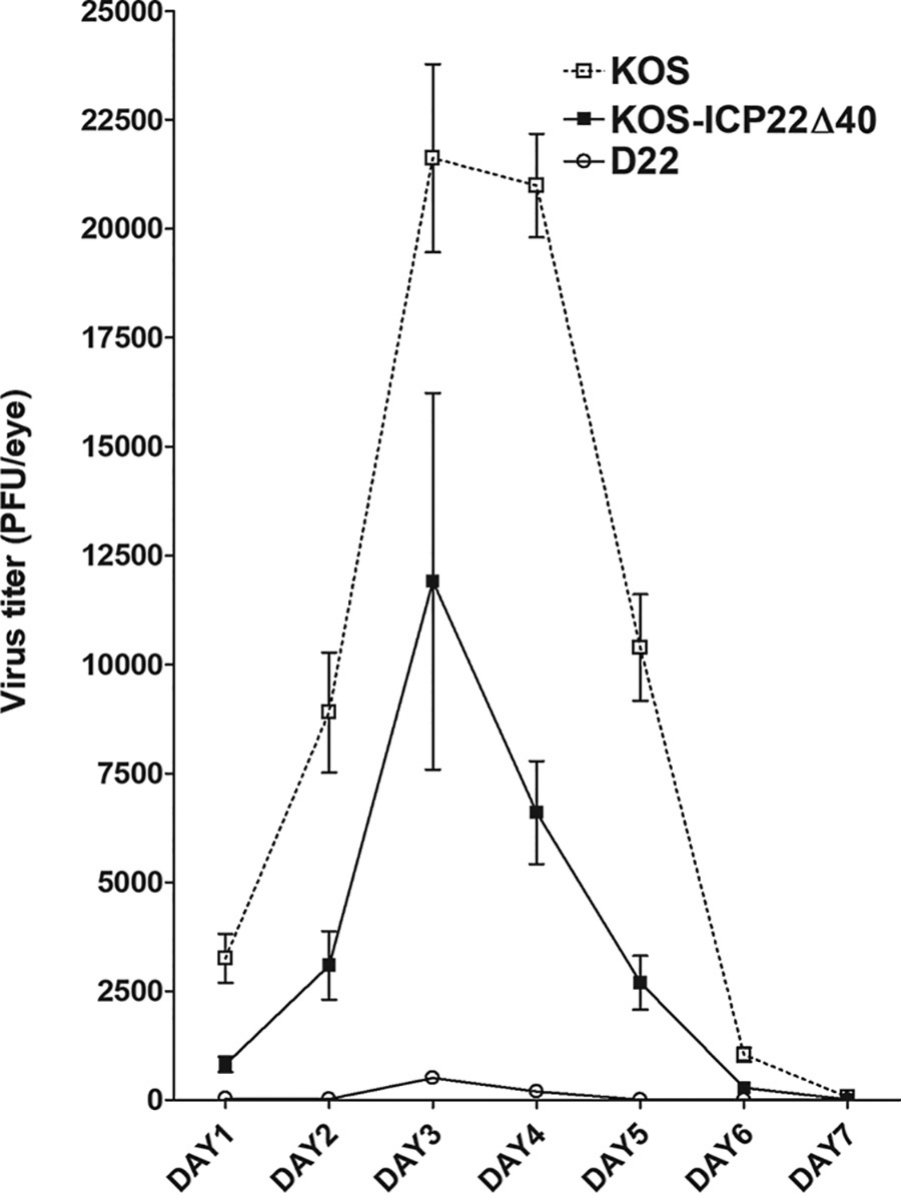
KOS-ICP22Δ40 replication in mouse tears after ocular infection. BALB/c mice were infected with 2 × 10^5^ PFU per eye of KOS-ICP22Δ40, D22 (KOS-ICP22Δ40 parental virus), or WT KOS (D22 parental virus) following corneal scarification. Tear films were collected daily on days 1 to 7, and virus titers were determined by standard plaque assays on Vero cells. Each point represents the mean titer ± SEM in tears from 30 eyes for KOS-ICP22Δ40 virus, 40 eyes for D22 virus, and 36 eyes for KOS virus.

To evaluate the effect of deleting ICP22 aa 305 to 345 on virus replication *in vivo*, BALB/c mice were infected ocularly with 2 × 10^5^ PFU/eye of the KOS-ICP22Δ40 mutant virus, WT KOS, or D22. Previously, we showed that D22 replicated at significantly lower levels in mouse eyes than WT KOS (29). Thus, tear films were collected from 30 to 40 eyes per group per time point, and virus titers were determined by plaque assays on Vero cells. Peak titers of all three viruses were found on day 3 p.i. (Fig. 5). From day 1 to day 6 p.i., virus titers in the eyes of WT-infected mice were significantly higher than those of either KOS-ICP22Δ40 or D22 virus (*P* < 0.01) (Fig. 5). Similarly, the KOS-ICP22Δ40 mutant virus had significantly higher virus replication on days 1 to 6 p.i. than D22 virus (*P* < 0.001) (Fig. 5). These results suggest that the insertion of the truncated *ICP22* gene into D22 virus partially rescued virus replication in the eyes of infected mice; however, WT levels of virus replication required full-length ICP22. These results are consistent with the lower levels of virus replication in tissue cultures described above (Fig. 4) and may not be associated with an immune response that reduces virus titers in tears.

### Similar levels of latency and reactivation in KOS-ICP22Δ40-infected mice and KOS-infected mice.

**FIG 6:**
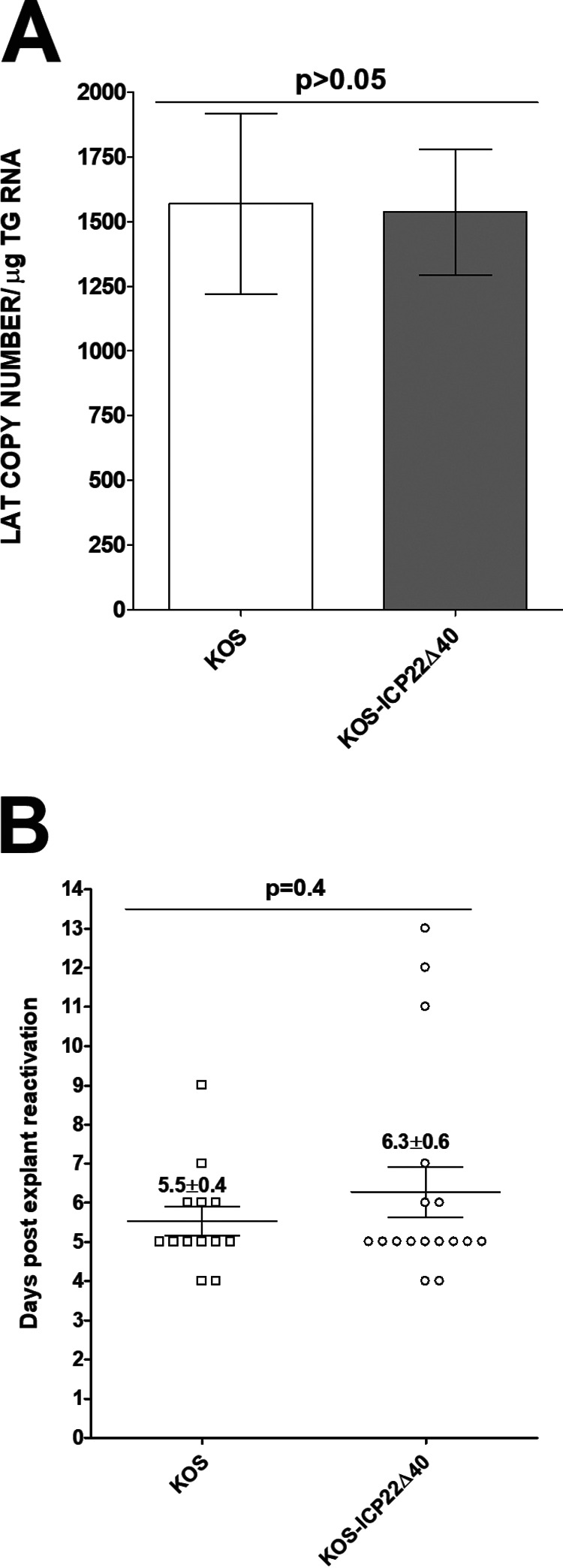
Latency and explant reactivation in TG of infected mice. (A) LAT levels in TG of latently infected mice. Following corneal scarification, BALB/c mice were ocularly infected with 2 × 10^5^ PFU/eye of KOS-ICP22Δ40 or WT KOS. On day 28 p.i., TG from latently infected mice were harvested, and qRT-PCR was performed on individual TG from each mouse. In each experiment, the estimated relative LAT copy number was calculated using standard curves generated from pGEM-5317 as we described previously (15, 29). GAPDH expression was used to normalize relative LAT RNA expression in the TG. Latency is based on 35 TG for KOS-ICP22Δ40 and 39 TG for KOS. *P* values were determined using one-way ANOVA. (B) Explant reactivation in TG of latently infected mice. BALB/c mice were ocularly infected as described above with each virus, and TG from latently infected mice were individually isolated on day 28 p.i. Each TG was individually incubated in 1.5 ml of tissue culture medium at 37°C. Medium aliquots were removed from each culture daily for up to 5 days and plated on indicator cells (RS cells) to assess the appearance of reactivated virus. Results are plotted as the number of TG that reactivated daily. Numbers indicate average times that the TG from each group first showed cytopathic effect (CPE) ± SEM. Reactivation is based on 18 TG for KOS-ICP22Δ40-infected mice and 13 TG for KOS-infected mice. *P* values were determined using one-way ANOVA.

Following corneal scarification, BALB/c mice were infected with 2 × 10^5^ PFU/eye of KOS-ICP22Δ40 or KOS virus. Individual TG from 18 to 20 infected mice were harvested on day 28 p.i., and latency levels were determined by real-time PCR or quantitative reverse transcription-PCR (qRT-PCR) based on HSV-1 latency-associated transcript (LAT) expression as described in Materials and Methods. Unlike the lower viral replication in the eyes of KOS-ICP22Δ40-infected than in WT KOS-infected mice (Fig. 5), latency was not enhanced in KOS-ICP22Δ40-infected mice, suggesting that the deletion of 40 aa of ICP22 does not alter the latency establishment and/or maintenance seen in WT KOS-infected mice (*P* > 0.05) (Fig. 6A). This is consistent with our previous study showing a lack of correlation between primary virus titers in the eyes and levels of viral latency in TG of latently infected mice (37).

We next tested explant reactivation from latency in mice ocularly infected with 2 × 10^5^ PFU/eye of HSV-1 KOS-ICP22Δ40 and KOS after corneal scarification. Virus reactivation was analyzed by explanting individual TG from 10 infected mice on day 28 p.i. as described in Materials and Methods. As with latency levels, mice infected with KOS-ICP22Δ40 or KOS virus had similar reactivation times (*P* > 0.05) (Fig. 6B). However, in contrast to primary virus replication levels *in vitro* and *in vivo*, the time to explant reactivation in KOS-ICP22Δ40-infected mice correlated with the latency levels.

### Upregulation of CD80 and IFN-γ in corneas of mice ocularly infected with KOS-ICP22Δ40 virus.

**FIG 7:**
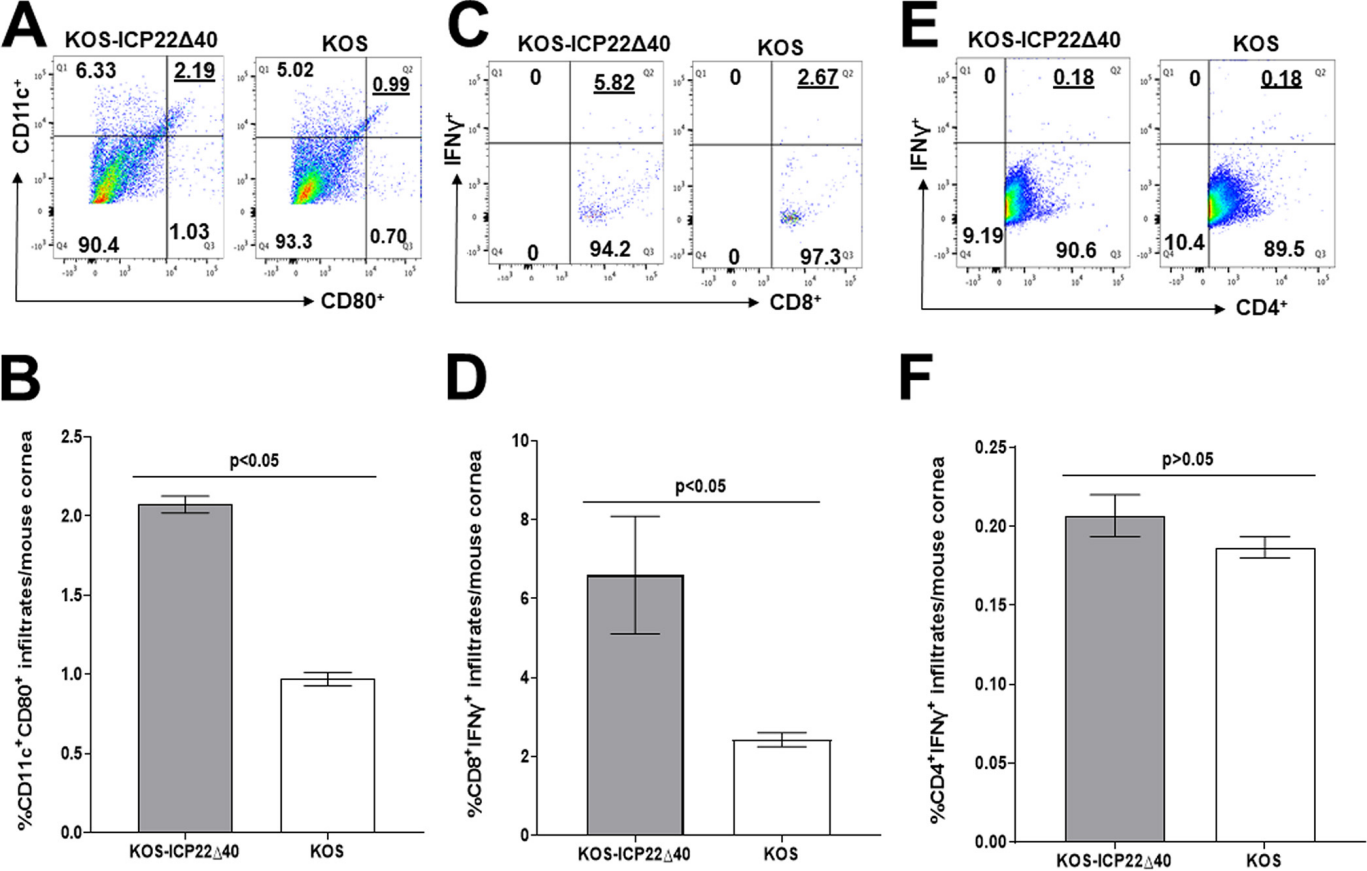
Increased CD11c^+^ CD80^+^ and CD8^+^IFN-γ^+^ infiltrates in corneas of mice infected with KOS-ICP22Δ40 virus. Mice were ocularly infected as described in the legend of Fig. 5 with KOS-ICP22Δ40 or WT KOS, and corneas of infected mice were harvested on day 5 p.i. Corneas from three mice for each virus were combined, and single-cell suspensions were stained with combinations of anti-CD11c, anti-CD4, anti-CD8, anti-CD80, or anti-IFN-γ and analyzed by flow cytometry. Experiments were repeated three times. Graphs represent average results from three independent experiments. Error bars represent SEM, and *P* values were calculated using Fisher’s exact test and considered significant for a *P* value of <0.05.

Previously, we reported that ICP22 suppresses CD80 expression *in vitro* and *in vivo* and that a recombinant HSV-1 lacking ICP22 upregulated CD80 and IFN-γ expression in the corneas of ocularly infected mice (15, 29). We have also shown that the overexpression of CD80 by a recombinant HSV-1 expressing CD80 (HSV-CD80) enhanced CD8 expression in the corneas of infected mice, leading to increased eye disease (15, 38, 39). In contrast to D22 recombinant virus, which lacks the entire *ICP22* gene (33), KOS-ICP22Δ40 recombinant virus lacks only 40 aa of ICP22 and can replicate more efficiently both *in vitro* and *in vivo* than D22 virus. Thus, to determine how the absence of ICP22 binding to the CD80 promoter affects CD80, CD4, CD8, and IFN-γ expression in the corneas of infected mice, following corneal scarification, we ocularly infected nine BALB/c mice with KOS-ICP22Δ40 virus or the WT KOS control virus. Corneas were isolated on day 5 p.i., and corneas from three mice per virus were combined. Single-cell suspensions were prepared and stained with combinations of anti-CD11c, anti-CD80, anti-CD4, anti-CD8, or anti-IFN-γ monoclonal antibodies. Fluorescence-activated cell sorter (FACS) analysis revealed that CD80 expression in DCs was higher in KOS-ICP22Δ40-infected mice than in KOS-infected mice (2.19% versus 0.99%) (Fig. 7A). The experiment was repeated twice, with results depicted in Fig. 7B. Thus, infection of mice with KOS-ICP22Δ40 virus resulted in significantly more CD11c^+^CD80^+^ expression in the corneas of infected mice than in mice infected with WT KOS virus (*P* < 0.05) (Fig. 7B). The percentage of CD8^+^IFN-γ^+^ cells was also higher in KOS-ICP22Δ40-infected mice than in KOS-infected mice (5.82% versus 2.67%) (Fig. 7C), and results from three independent experiments are shown in Fig. 7D. KOS-ICP22Δ40 infection resulted in significantly more CD8^+^IFN-γ^+^ cells in the corneas of infected mice than in those from WT KOS virus-infected mice (*P* < 0.05) (Fig. 7D). The percentages of CD4^+^IFN-γ^+^ cells in KOS-ICP22Δ40-infected corneas were similar to those in parental virus-infected corneas (0.18% versus 0.18%) (Fig. 7E) and were not statistically different (*P* > 0.05) (Fig. 7F). Thus, as expected, and similar to ICP22-null virus, the absence of ICP22 binding to the CD80 promoter increased both CD80 and CD8^+^IFN-γ^+^ expression but not CD4^+^IFN-γ^+^ expression. Therefore, increased CD8^+^IFN-γ^+^ cell numbers but not CD4^+^IFN-γ^+^ cell numbers correlated with increased CD80 expression by KOS-ICP22Δ40 virus. This is consistent with our results following infection of mice with a recombinant virus expressing CD80 (15, 38).

## DISCUSSION

Our previous studies demonstrated that (i) HSV-1 ICP22 downregulates the expression of CD80 but not that of CD86, in the presence or absence of anti-HSV-1 antibodies (15); (ii) a recombinant HSV-1 expressing CD80 exacerbated CS in infected BALB/c and C57BL/6 mice (15, 40); (iii) the suppression of CD80 by ICP22 is mediated by the direct binding of HSV-1 ICP22 to the CD80 promoter (15); (iv) mice ocularly infected with a recombinant HSV-1 lacking ICP22 developed enhanced eye disease (29); (v) the expression of CD80 by HSV-1 in place of LAT compensated for the latency reactivation and antiapoptotic functions of LAT (40); and (vi) CD80 plays a critical role in increased inflammatory responses in HSV-1-infected mouse corneas (29, 38). Thus, suppression of CD80 expression by ICP22 may be a mechanism of virus self-survival and immune escape. Collectively, our goals in this study were to (i) fine map the ICP22 amino acids involved in binding to the CD80 promoter, (ii) construct a recombinant virus that expresses ICP22 but does not bind to the CD80 promoter and thus does not suppress CD80 expression *in vitro* and *in vivo*, and (iii) compare the immunogenicities of this recombinant virus and the WT virus relative to primary HSV-1 infection, immune infiltrates in the eye, and latency reactivation in ocularly infected mice.

ICP22 and CD80 are conserved genes (27, 30), and based on our published results (29, 38, 40), we tested the hypothesis that the interruption of ICP22 binding to the CD80 promoter will affect virus infectivity *in vitro* and *in vivo*. ICP22 is one of the five HSV-1 immediate early genes, and the initial steps of replication begin with these five ICP proteins (31, 32). ICP22 is indispensable for virus replication *in vivo* but not *in vitro* (41). However, the replication of ICP22-null virus *in vitro* is host cell dependent, and virus grows efficiently in Vero cells but not in RS cells (33). ICP22 contains 420 amino acids (30, 33). To test our overall hypothesis, we first mapped the ICP22 amino acids required for binding to the CD80 promoter using overlapping ICP22 fragments. We mapped the amino acids required for ICP22 binding to the CD80 promoter to ICP22 aa 305 to 345 and used these results to construct an ICP22 recombinant virus lacking aa 305 to 345 of ICP22 such that the truncated ICP22Δ40 protein contained the original 380 amino acids. This recombinant virus grew more slowly than the control virus *in vitro* and in the eyes of infected mice; however, it grew significantly faster than ICP22-null virus, as we and others have reported previously (29, 33). Low expression levels of ICP22 in the mutant virus may have contributed to its phenotype *in vivo* compared with the WT virus.

To determine the expression of truncated ICP22 in ICP22Δ40, we used Western blot analysis. The 40-amino-acid region that was deleted from aa 305 to 345 of ICP22 migrated slightly faster than ICP22 from WT KOS. The faster-migrating band of the two observed in KOS virus is the US1.5 gene that has been observed and reported previously (33, 36). The US1.5 gene (aa 90 to 420) has an ATG site at aa 90 of ICP22 and after the full-length ICP22 start site. Both genes end at the same point. The level of ICP22Δ40 protein expression was significantly lower than that of KOS, which supports our observation that the level of *in vitro* replication of ICP22Δ40 virus was significantly lower than that of KOS virus replication. Furthermore, our *in vivo* studies of eye swabs from ICP22Δ40-infected mice showed less virus replication than in KOS-infected mice. US1.5 protein expression, which is visible in the KOS lane, was below the detection limit for ICP22Δ40 virus, which could be due to its lower infectivity. Interestingly, our study showed an abatement of ICP22Δ40 virus replication (Fig. 5) between KOS and D22 virus titers, which mirrors the results of Mostafa and Davido (33), in which a mutant virus lacking full-length ICP22 but expressing US1.5 has a similar replication profile, which is also intermediate between KOS and D22. Taken together, the expression of recombinant ICP22Δ40 virus restricted viral infectivity and curbed the replication rate below that of KOS but higher than that of D22 virus.

In this study, we demonstrated that KOS-ICP22Δ40 virus significantly enhanced the expression of CD80 by CD11c in the corneas of ocularly infected mice relative to WT virus. These results are consistent with our previous study showing that infection of mice with WT virus suppressed CD80 expression in DCs (15), while the absence of ICP22 in ICP22-null virus enhanced the expression of CD80 in DCs in infected mouse corneas (29). CD80 is expressed by several different cell types (42). Our previous studies showed that HSV-1 infection downregulates CD80 expression by DCs irrespective of the source of DCs and does not affect CD80 expression by macrophages, T cells, or monocytes (15, 29). In addition, in this study, we showed that the absence of ICP22 binding to the CD80 promoter increased the level of IFN-γ expression in CD8^+^ T cells. Previously, we have shown that a recombinant HSV-1 expressing CD80 also increased CD8 expression in infected mouse corneas, leading to higher levels of eye disease (15, 38). Furthermore, the higher expression levels of CD8^+^IFN-γ^+^ cells in the corneas of KOS-ICP22Δ40-infected mice are not expected to reduce virus replication in the eyes of infected mice. Despite having 380 of its 420 amino acids, this recombinant virus grew less efficiently in RS and Vero cells than the parental WT virus. However, in this study, the upregulation of CD80 expression did not affect CD4 expression relative to WT virus. Our previous study showed that mice infected with recombinant HSV-1 expressing murine CD80 exacerbated corneal scarring (15). These results confirm our previous results showing that HSV-1 uses ICP22 to suppress CD80 expression and that this suppression plays a critical role in the immune evasion of HSV-1 while simultaneously protecting the host from HSV-1-induced pathology. This study further confirms our results that none of the more than 85 HSV-1 genes and remaining known immediate early genes (i.e., *ICP4*, *ICP27*, and *ICP47*) play any role in CD80 suppression.

Despite KOS-ICP22Δ40 virus replicating with lower kinetics *in vitro* and *in vivo*, mice infected with this virus had levels of latency and reactivation similar to those of the WT virus. This is likely a consequence of immune escape due to a lack of ICP22 binding to the CD80 promoter. The lack of a correlation between viral load, latency, and reactivation is probably due to higher CD80 expression levels. However, the positive correlation between latency and reactivation is consistent with our previous studies (37). It is possible that increased latency reactivation could be due to higher CD80 expression levels by DCs seen in this study, as we have previously reported that DCs drive latency and reactivation (43–45).

In conclusion, we have confirmed and extended our previous findings that ICP22 plays a pivotal role in HSV-1 immune escape by suppressing CD80 expression. Thus, the results generated by this study will potentially establish a previously undescribed mechanism underlying viral immune evasion that could be exploited to better manage HSV infection. In light of recent failures of several large-scale phase III HSV-1 vaccine trials, if we can develop a way to interfere with this interaction, it may provide a new strategy to reduce the inflammatory responses and inflammation associated with HSV infection.

## MATERIALS AND METHODS

### Ethics statement.

All animal procedures were performed in strict accordance with the Association for Research in Vision and Ophthalmology statement for the use of animals in ophthalmic and vision research and the NIH *Guide for the Care and Use of Laboratory Animals* (46). The animal research protocol was approved by the Institutional Animal Care and Use Committees of Cedars-Sinai Medical Center (protocol numbers 5030 and 8837).

### Viruses, cells, and mice.

Triply plaque-purified WT KOS (parental virus for D22), D22 (parental virus for KOS-ICP22Δ40), and KOS-ICP22Δ40 viruses were used in this study and grown as we described previously (39). The D22 recombinant virus, with *ICP22* deleted, was described previously (29, 33). Rabbit skin (RS) cells were grown in Eagle’s minimal essential medium (EMEM) supplemented with 5% fetal calf serum (FCS). Vero cells obtained from the American Type Culture Collection (ATCC) were grown in Dulbecco’s modified Eagle’s medium supplemented with 5% fetal bovine serum (FBS) as described previously (33). Throughout this article, HEK 293 cells (ATCC) are referred to as 293 cells. For transfections using 293 cells, cells were cultured in EMEM supplemented with 10% FBS as we described previously (15). Cells were typically passaged at 70 to 80% confluence and grown in a 37°C incubator at 5% CO_2_. Female 6-week-old inbred BALB/c mice (The Jackson Laboratory, Bar Harbor, ME) were used.

### Construction of the truncated ICP22 plasmid.

The truncated ICP22 plasmid was constructed using sequences of HSV-1 strain KOS (34). We synthesized a plasmid containing the *ICP22* gene with a 40-aa deletion corresponding to aa 305 to 345 of *ICP22*. This construct also contained 463 bp of KOS sequences upstream of the *ICP22* ATG and 554 bp downstream of the *ICP22* termination codon. The resulting plasmid, designated pICP22Δ40, contains 1,152 bp of the truncated *ICP22* gene bounded by 463- and 554-bp adjacent regions of *ICP22* sequences.

### Construction of KOS-ICP22Δ40 virus.

The parental virus for the KOS-ICP22Δ40 construct was D22, a mutant of the HSV-1 KOS strain in which the *ICP22* gene was completely deleted. D22 virus grows in Vero cells but not in RS cells (33). KOS-ICP22Δ40 virus was generated by homologous recombination as we previously described (47). Briefly, pICP22Δ40 DNA and infectious D22 DNA were cotransfected into RS cells using the calcium phosphate method. Cotransfected cells were plated, isolated plaques were picked, and DNA from isolated plaques was screened for the insertion of the truncated *ICP22* gene by PCR using ICP22 forward (GCGCCACCTGATACGCGACTG) and reverse (CGACCGCAGACAGCCAGGGC) primers. The PCR product length for WT ICP22 was 458 bp, and that for truncated ICP22 was 338 bp. All selected plaques were verified as having 338-bp amplified products, and a single plaque chosen for purification was designated KOS-ICP22Δ40.

### Plasmids.

Previously, we synthesized 759 bp of the CD80 promoter and inserted it into the multiple-cloning site of pGL4 to drive the expression of luciferase under the control of the CD80 promoter (15). We refer to these plasmids as pGL4-80 plasmids. We also previously synthesized ICP22-FLAG (GenScript, Piscataway, NJ) (15) containing the complete open reading frame (ORF) of the *ICP22* gene (1,260 bp) and two copies of FLAG sequences inserted into the BamHI site of pcDNA3.1. We also synthesized *ICP22* fragments corresponding to different regions of *ICP22* (Fig. 2). Similar to full-length *ICP22*, these constructs all have an ATG, two copies of the FLAG sequence, and a termination codon inserted into the pcDNA3.1 BamHI site.

### Transfection.

Transfection experiments were conducted using 293 cells and Gene Porter 2 (Genlantis, San Diego, CA) as we described previously (15). Briefly, cells were grown to 70 to 80% confluence in 12-well plates. Immediately before the experiment, plasmids were diluted in the dilution buffer provided, and transfection reagents were resuspended in EMEM (no FBS) in individual tubes. The reagents were then combined, incubated for 5 min, and added to the plates. Cells were transfected with either the promoterless luciferase plasmid pGL4-EV or pGL4-CD80p, containing 759 bp of the CD80 promoter cloned into the pGL4 multiple-cloning sites to drive the expression of the luciferase reporter. pRL-SV40 (catalog number E2231; Promega), a *Renilla* luciferase reporter plasmid, was used as the cotransfected internal control to monitor baseline cell responses to transfection (10 ng/reaction). Using a dual-luciferase reporter system (Promega, Madison, WI), two individual reporters were introduced simultaneously to determine responses within the same cells. Sample preparation was conducted as described by the manufacturer (Promega). Cells were washed with phosphate-buffered saline (PBS) and lysed in lysis buffer, and the collected supernatants were transferred to 96-well plates. The luminometer (Glomax; Promega, Madison, WI) was primed with luciferase and Stop & Glow reagents. Assays were conducted in replicates of 10, and means ± standard errors of the means (SEM) were calculated from three separate experiments (*n* = 30).

### Virus replication in tissue culture.

RS cell monolayers at 70 to 80% confluence were infected with 0.1 or 1 PFU/cell of WT KOS or KOS-ICP22Δ40. Virus was harvested at 24 and 48 h postinfection by two cycles of freeze-thawing of the cell monolayers with medium. Virus titers were determined by standard plaque assays on Vero and RS cells.

### Ocular infection of mice.

BALB/c mice were infected ocularly with 2 μl of tissue culture medium containing 2 × 10^5^ PFU/eye of HSV-1 strain KOS, KOS-ICP22Δ40, or D22 with corneal scarification as we described previously (29). Before corneal scarification and ocular infection, mice were anesthetized with ketamine and dexmedetomidine. Following anesthesia and ocular infection, buprenorphine was administered by subcutaneous injection. Buprenorphine was administered again the morning after infection.

### Titration of virus in tears of infected mice.

Tear films were collected from both eyes of 15 to 20 mice per group on days 1 to 7 p.i. using a Dacron-tipped swab as described previously (48). Each swab was placed in 1 ml of MEM tissue culture medium and squeezed, and the amount of virus was determined using a standard plaque assay on Vero cells.

### *In vitro* explant reactivation assay.

Mice were sacrificed on day 28 p.i., and individual TG were removed and cultured in tissue culture medium as we described previously (15, 29, 40). Aliquots of medium were removed from each culture daily and plated on indicator RS cells to detect reactivated virus. Because medium from explanted TG cultures was plated daily, we could determine the time at which reactivated virus first appeared in explanted TG cultures.

### Detection of LAT RNA by quantitative RT-PCR analyses.

RNA was extracted from latent TG as we described previously (45, 49, 50). Quantitative RT-PCR analyses were performed using LAT-specific primers (forward primer 5′-GGGTGGGCTCGTGTTACAG-3′, reverse primer 5′-GGACGGGTAAGTAACAGAGTCTCTA-3′, and probe 5′-FAM [6-carboxyfluorescein]-ACACCAGCCCGTTCTTT-3′). The amplicon length for the LAT primer set is 81 bp, corresponding to LAT nucleotides (nt) 119553 to 119634. Relative LAT RNA copy numbers were calculated using standard curves generated from the pGem5317 plasmid as we described previously (39, 51). In all experiments, glyceraldehyde-3-phosphate dehydrogenase (GAPDH) was used to normalize transcripts.

### Western blot analysis.

293 cells were grown in 10-cm plates and infected with 1.5 PFU/cell of KOS, D22, or ICP22Δ40 or mock infected. One hour after the addition of virus, medium in the cell culture plate was removed, and cells were washed with PBS and replated with fresh medium. Infected or mock-infected cells were harvested at 5 h postinfection, and cells were harvested, lysed with radioimmunoprecipitation assay (RIPA) buffer, and centrifuged in microcentrifuge tubes at 14,000 rpm for 15 min. The supernatant of each lysate was transferred to a new tube and kept on ice. Lysate protein concentrations were measured using a reducing-agent-compatible bicinchoninic acid (BCA) protein assay kit (Pierce Chemical, Dallas, TX). We loaded 40 μg of the total whole-cell lysate onto each lane of a 10% SDS-PAGE gel for electrophoresis. The blot was probed overnight using primary anti-ICP22 at 1:5,000, rabbit polyclonal antibody 413 (Bethyl Laboratories, Montgomery, TX), and β-actin monoclonal rabbit antibody at 1:5,000 (Cell Signal Technology, Danvers, MA). For detection, a secondary anti-rabbit IgG horseradish peroxidase (HRP)-linked antibody was diluted at 1:4,000 (Cell Signal Technology, Danvers, MA) and incubated for 2 h. Polyvinylidene difluoride (PVDF) membranes (MilliporeSigma, Burlington, MA) were washed three times with 1× Tris-buffered saline–Tween (TBS-T) and developed using Signalfire ECL (Cell Signal Technology).

### FACS analysis.

After corneal scarification, BALB/c mice were infected with 2 × 10^5^ PFU/eye of KOS-ICP22Δ40 or WT KOS virus. Corneas from three mice were combined, and single-cell corneal suspensions were prepared as we described previously (37, 52, 53). After washing the cell suspension, pelleted cells were resuspended in cell culture medium that was treated with brefeldin A for 1 h (catalog number 420061; BioLegend, San Diego, CA). Single-cell corneal suspensions were stained with combinations of anti-CD4 (catalog number 560782, anti-mouse V500, clone RM4-5), anti-CD8α (catalog number 100706, anti-mouse fluorescein isothiocyanate [FITC], clone 53-6.7), anti-IFN-γ (catalog number 505810, anti-mouse allophycocyanin [APC], clone XMG1.2), anti-CD11c (catalog number 117330, brilliant violet 421 anti-mouse, clone N418), or anti-CD80 (catalog number 104708, phycoerythrin [PE] anti-mouse, clone 16-10A1) monoclonal antibodies (BD Biosciences, San Jose, CA, and BioLegend) and analyzed by flow cytometry as we described previously (29, 54). Experiments were repeated three times using a total of nine mice per virus. Stained cells were analyzed using a BD LSR II flow cytometer and BD FACSDiva software (BD Biosciences). Postexperiment data analysis was performed using FlowJo software v10.7.1 (BD Biosciences).

### Statistical analysis.

Data were analyzed by Student’s *t* test, Fisher’s exact test, and analysis of variance (ANOVA) using GraphPad v9 (GraphPad, San Diego, CA). Results were considered statistically significant if the *P* value was <0.05.
